# Participation in Sports Organizations and the Prevention of Functional Disability in Older Japanese: The AGES Cohort Study

**DOI:** 10.1371/journal.pone.0051061

**Published:** 2012-11-30

**Authors:** Satoru Kanamori, Yuko Kai, Katsunori Kondo, Hiroshi Hirai, Yukinobu Ichida, Kayo Suzuki, Ichiro Kawachi

**Affiliations:** 1 Faculty of Health and Care and Nursing, Juntendo University, Tokyo, Japan; 2 Physical Fitness Research Institute Meiji Yasuda Foundation of Health and Welfare, Hachioji, Japan; 3 Center for Well-being and Society, Nihon Fukushi University, Nagoya, Japan; 4 Faculty of Engineering, Department of Civil and Environmental Engineering, Iwate University, Morioka, Japan; 5 Department of Society, Human Development, and Health, Harvard School of Public Health, Boston, Massachusetts, United States of America; Universidad Europea de Madrid, Spain

## Abstract

**Background:**

We sought to examine prospectively the difference in the association between incident functional disability and exercise with or without sports organization participation.

**Methods:**

The study was based on the Aichi Gerontological Evaluation Study (AGES) Cohort Study data. In October 2003, self-reported questionnaires were mailed to 29,374 non-disabled Japanese individuals aged 65 years or older. Of these, 13,310 individuals were introduced to the Study, and they were followed for 4 years. Analysis was carried out on 11,581 subjects who provided all necessary information for the analysis.

**Results:**

Analysis was carried out on incident functional disability by 4 groups of different combinations of performance of exercise and participation in a sports organization Active Participant (AP), Exercise Alone (EA), Passive Participant (PP) and Sedentary (S). Compared to the AP group, the EA group had a hazard ratio (HR) of 1.29 (1.02–1.64) for incident functional disability. No significant difference was seen with the PP group, with an HR of 1.16 (0.76–1.77). When a measure of social networks was added to the covariates, the HR of the EA group dropped to 1.27 (1.00–1.61), and significant differences disappeared. In contrast, it showed hardly any change when social support was added.

**Conclusion:**

The results suggested that, even with a regular exercise habit, incident functional disability may be better prevented when a person participates in a sports organization than when he/she does not. In addition, participation in a sports organization correlates positively with social networks, which may lead to a small decrease in incident functional disability.

## Introduction

Over 1 in 5 people are 60 years or older in more developed regions today, and aging is predicted to advance in developing nations as well in the future, [1]. At 31.1% of the country’s population, Japan has the largest proportion of people who are 60 years and older in the world, and the number of older people with incident functional disability is increasing as society continues to age, [2]. Reducing this number is an urgent task throughout the world.

Exercise is an effective way to prevent incident functional disability. Training programs that include special exercises have been reported to influence numerous factors related to functional disability, such as maintenance of physical functioning, [3], prevention of falls, [4] and improvement of cognitive functioning, [5]. Such special exercise training programs may not even be necessary; as links have also been shown between walking and physical activity that includes exercise, and a decrease in functional disability, [6]–[11].

While people may exercise alone, they may also join a group or organization to get exercise. Mechanisms for the effect of participation in organized sports on health may include not only physiological mechanisms through the increase in physical activity, but also mechanisms whereby social networks and social support are more easily obtained through the joining of a group or organization, [12]. Links have been shown between poor social relationships and a decline in functional status, [7] as well as death, [13], [14]. This means that, in addition to physiological effects of exercise, getting exercise by participating in a sports organization may have additional effects from social relationships which are not achieved in exercise without participation in an organization. To the best of our knowledge, the effect of the latter has not been tested in any studies to date. If participation in a sports organization is indeed more strongly linked to incident functional disability prevention than private exercise, approaches for incident functional disability prevention should involve increasing participation in a sports organization in addition to recommending private exercise.

This prospective cohort study of older Japanese people aimed to test the relationship between incident functional disability and differences in whether or not subjects exercised and/or participated in a sports organization.

## Methods

### Study Sample

The present study is based on the Aichi Gerontological Evaluation Study (AGES) Cohort Study data, [15], [16]. This study involves investigating factors associated with incident functional disability among non-institutionalized elderly individuals aged 65 years or older. The region studied covered 6 municipalities in the Chita-hanto Peninsula of Aichi Prefecture, Japan (Handa city, Tokoname city, Agui town, Taketoyo town, Minamichita town and Mihama town). In October 2003, self-reported questionnaires were mailed to 29,374 community-dwelling individuals aged 65 years or older who were not eligible to receive benefits from public long-term care insurance (LTCI) services. The survey was conducted using a random sampling method in the 2 larger municipalities (Handa city and Tokoname city) and a complete census (complete enumeration) of the 4 smaller municipalities (Agui town, Mihama town, Minami-Chita town, and Taketoyo town) by municipal officers of the public LTCI system. The official residential registries were maintained by the municipal administrations, and the Japanese registries included information such as age. Questionnaires were sent to 5,000 people each from Handa city and Tokoname city and to all eligible people in the other municipalities. Of those, 13,310 individuals (6,508 males; 6,802 females) were introduced to the AGES Cohort. They were followed for a 4-year period starting in November 2003 (observation period: November 2003 to October 2007). Analysis was carried out on 11,581 subjects, excluding 319 people whose information on age or sex was missing, and 1,410 people who did not respond to questions on frequency of exercise and participation in a sports organization. Subjects were 5,700 males (49.2%) and 5,881 females (50.8%), and the mean age was 72.6±6.1 years. Baseline characteristics of the participants have been reported elsewhere, [15], [16].

Ethical approval for the study was obtained from the Nihon Fukushi University Ethics Committee.

### Incident Functional Disability

We defined the state of becoming eligible for certification of needed long-term care within the procedure prescribed in the LTCI system that has been in place in Japan since 2000 as “incident functional disability.” Certification of needed long-term care is based on evaluation of the need for long-term care according to uniform criteria for all of Japan and based on both a home-visit interview and a written opinion from the primary physician, [17]. We obtained information on certification of needed long-term care, death, and moving out of the study area from the LTCI database maintained by the municipalities. The day certification of needed long-term care was issued was the application date for certification of needed long-term care.

### Performing Exercise and Participation in a Sports Organization

To define exercise, subjects were asked “Do you engage in any leisure activities at the moment?” Those who answered “Yes” were then asked about the frequency of performing a sports activity such as ground golf, gateball [Japanese croquet], walking, jogging or any other physical exercises (“frequency of exercise”). Subjects who responded with “almost every day,” “twice or three times a week,” or “once a week” were labeled “Exercisers,” and those who responded with “once or twice a month,” “several times a year” or “I don’t engage in any sports activities” were labeled as “Inactive.” Those who responded with “No” to the first question were considered the same as those who responded with “I don’t engage in any sports activities.” To determine participation in a sports organization, subjects were asked if they are a member of a “sports group or club.” Those who answered “Yes” were labeled “Participants” and those who answered “No” were labeled “Non-participants.”

### Covariates

Based on previous studies, [8]–[11], [18], age, sex, annual equivalent income, educational attainment, marital status, occupational status, self-reported medical conditions, depression (Geriatric Depression Scale: GDS), [19], smoking and alcohol consumption were used as covariates that may correlate with participation in a sports organization, performance of exercise and incident functional disability. Social networks and social support were used to test which aspect of participation in a sports organization accounts for the prevention of incident functional disability, since previous studies indicate that social relations are important mediating factors in the mechanisms for the effect of participation in organized sports on health, [12]. Frequency of meeting friends was used as a measure of social networks, and social support was measured with four types: “receiving” and “providing” emotional and instrumental support.

**Table 1:** Combination of frequency of exercise and participation in a sport organization.

		Sport organization	
		Participation	Non-participation
Exercise	Once a monthor more	Active Participant(AP)	Exercise Alone(EA)
	Less than oncea month	Passive Participant(PP)	Sedentary(S)

### Statistical Analysis

As shown in Table 1, subjects were first split into 4 groups based on whether or not they performed exercise and participated in a sports organization. Table 2 shows baseline characteristics and the incident rate of functional disability over 4 years for each group related to performance of exercise and participation in a sports organization. To test for group differences, one-way analysis of variance (ANOVA) was performed on age, and χ2 tests were performed on sex, frequency of exercise, social networks and social support. Next, Cox’s proportional hazards model was used to calculate the hazard ratio (HR) of incident functional disability over 4 years. Respondents who were lost to follow-up by moving or death without incident functional disability, were included as censored data in the models. Regression analysis was performed with simultaneous forced entry of age, sex, annual equivalent income, educational attainment, marital status, occupational status, self-reported medical conditions, depression, smoking and alcohol consumption as covariates (Model 1).

**Table 2:** Baseline characteristics.

		Active Participant (AP)	Exercise Alone (EA)	Passive Participant (PP)	Sedentary (S)	p
N		1,888	2,548	447	6,698	–
Age (years)	Mean±SD	70.7±4.9	71.8±5.5	70.7±5.0	73.5±6.6	<.001
		%				
Sex (%)	Males	52.2	57.1	55.7	45.0	<.001
Frequency of exercise (%)	Almost everyday	28.1	57.1	0.0	0.0	<.001
	Twice or three times a week	44.5	28.4	0.0	0.0	
	Once a week	27.3	14.6	0.0	0.0	
	Once or twice a month	0.0	0.0	31.3	2.6	
	Several times a year	0.0	0.0	3.8	1.1	
	Never	0.0	0.0	64.9	96.3	
Frequency of meeting friends (%)	Once a month or more	93.3	75.5	87.0	65.7	<.001
	Less than once a month	5.7	23.0	10.5	30.4	
	Missing	1.0	1.5	2.5	4.0	
Receiving emotional support (%)	Yes	90.3	86.8	86.8	82.9	<.001
	No	6.5	8.9	9.8	10.7	
	Missing	3.2	4.3	3.4	6.4	
Providing emotional support (%)	Yes	86.3	81.6	83.4	73.8	<.001
	No	9.7	13.6	12.3	18.3	
	Missing	4.0	4.8	4.3	7.9	
Receiving instrumental support (%)	Yes	94.0	91.1	92.6	88.9	<.001
	No	3.8	5.4	4.5	5.8	
	Missing	2.2	3.5	2.9	5.3	
Providing instrumental support (%)	Yes	92.5	90.0	92.2	83.5	<.001
	No	3.8	5.7	4.3	9.1	
	Missing	3.7	4.2	3.6	7.3	

* P-value was calculated using one-way analysis of variance (ANOVA) for age.

* P-values were calculated using χ^2^ test for sex, frequency of frequency of exercise of meeting friends, receiving emotional support, providing emotional support, receiving instrumental support, and providing instrumental support.

To test which aspect of participation in a sports organization accounts for the prevention of incident functional disability, we added one social network/support measure to each model from Model 2 to Model 6 and inspected the change in the HR estimate associated with sports participation. Thus, in Mode1 2 for example, we added the variable “frequency of meeting friends” (as a measure of social network) above and beyond the variables in Model 1. In a similar manner, we added the following additional variables in subsequent models: receiving emotional support in Model 3, providing emotional support in Model 4, receiving instrumental support in Model 5, and providing instrumental support in Model 6.

All variables except for age were set as dummy variables. A “missing” category was used in analysis to account for missing values in response to questions regarding the covariates. SPSS 18.0J was used for statistical analysis with a significance level of 5%.

## Results

Table 2 shows baseline characteristics. A total of 4,436 subjects exercised once or more a week (38.3%) and 2,335 subjects (20.2%) participated in a sports organization.

There were 1,888 subjects (16.3%) in the Active Participant (AP) group, 2,548 subjects (22.0%) in the Exercise Alone (EA) group, 447 subjects (3.9%) in the Passive Participant (PP) group and 6,698 subjects (57.8%) in the Sedentary (S) group. Mean age was lowest in the AP group and highest in the S group, with a difference of 2.8 years. The proportion of males was below 50% only in the S group. Regarding frequency of exercise, nearly twice the people in the EA group exercised “almost every day” compared to the AP group. Also, more than ten times more people in the PP group exercised “once or twice a month” compared to the S group. The ratio of subjects who met friends once a month or more decreased in order of AP, PP, EA and S, showing a trend for greater frequency of meeting friends by those who participated in a sports organization. Social support showed the same pattern as frequency of meeting friends, with the ratio of subjects who said they have social support decreasing in order of AP, PP, EA and S for all aspects of support except for receiving emotional support. However, the difference between groups was smaller than that for frequency of meeting friends.

Among the 11,581 subjects analyzed, 909 people died (331 people developed an incident functional disability before they died), 1,380 people developed an incident functional disability and 128 people moved out of the research area during the 4 year follow-up period. The incident rate of functional disability was calculated by dividing the person-years of observation from the number of people who developed an incident functional disability (Table 3). Incident rate was lowest in the AP group, followed by the PP group, the EA group and the S group, in increasing order. The same trend was seen when the data was stratified by age.

**Table 3:** Incident rate of functional disability for 4 years.

		Active Participant (AP)	Exercise Alone (EA)	Passive Participant (PP)	Sedentary (S)
	Age(years)				
N (%)	65–74	1,503(79.6)	1,815(71.2)	340(76.1)	3,681(55.0)
	75+	385(20.4)	733(28.8)	107(23.9)	3,017(45.0)
	total	1,888(100.0)	2,548(100.0)	447(100.0)	6,698(100.0)
Incident/Person year	65–74	39/6423	77/7850	10/1491	201/16776
	75+	61/845	148/1661	18/228	826/7145
	total	100/7268	225/9511	28/1719	1027/23921
Incident rate	65–74	0.006	0.010	0.007	0.012
	75+	0.072	0.089	0.079	0.116
	total	0.014	0.024	0.016	0.047

Table 4 shows the results of analyzing incident functional disability by performance of exercise and participation in a sports organization using Cox’s proportional hazards model. Setting the “Exerciser” group as the reference, the HR for the “Inactive” group was significantly high at 1.26 (95% confidence intervals: 1.10–1.45). Setting the “Participant” group as the reference, the HR for the “Non-participant” group was also significantly high at 1.33 (1.09–1.62).

**Table 4:** Adjusted hazard ratios (95% confidence intervals) for incident functional disability by exercise and participation in a sport organization.

	N	Crude HR (95% CI)	Adjusted HR (95% CI)
Exerciser	4,436	1.00	1.00a)
Inactive	7,145	2.13(1.88–2.42)	1.26(1.10–1.45)
Participant	2,335	1.00	1.00b)
Non-participant	9,246	2.64(2.20–3.17)	1.33(1.09–1.62)

a) Adjusted for age, sex, annual equivalent income, educational attainment, marital status, occupation status, self-reported medical conditions, depression, smoking, alcohol consumption, and participation in a sport organization.

b) Adjusted for age, sex, annual equivalent income, educational attainment, marital status, occupation status, self-reported medical conditions, depression, smoking, alcohol consumption, and exercise.

Table 5 shows the results of analyzing incident functional disability by the 4 groups of different combinations of performance of exercise and participation in a sports organization using Cox’s proportional hazards model. Setting the AP group as the reference, the HR for the EA group was significantly high at 1.29 (1.02–1.64) and was even higher for the S group at 1.65 (1.33–2.04). No significant difference was seen in the PP group, with an HR of only 1.16 (0.76–1.77). As it is likely that subjects who responded that they participated in a sports organization but that they “Never” exercised also hardly ever participated in their sports organization, we then conducted sub-analysis with these subjects in the “S” group. The number of subjects in the PP group dropped from 447 people to 157 people, but the HR only changed from 1.16 (0.76–1.77) to 1.15 (0.56–2.37) and the lack of a difference between the PP group and the AP group was therefore maintained.

**Table 5:** Adjusted hazard ratios (95% confidence intervals) for incident functional disability by combination of exercise and participation in a sport organization.

	N	Crude HR (95% CI)	Adjusted HR (95% CI) Model 1a)
Active Participant (AP)	1,888	1.00	1.00
Exercise Alone (EA)	2,548	1.72(1.36–2.18)	1.29(1.02–1.64)
Passive Participant (PP)	447	1.18(0.78–1.80)	1.16(0.76–1.77)
Sedentary (S)	6,698	3.14(2.56–3.85)	1.65(1.33–2.04)

a) Model 1 is adjusted for age, sex, annual equivalent income, educational attainment, marital status, occupation status, self-reported medical conditions, depression, smoking, and alcohol consumption.

Next, social networks and social support were used to test which aspect of participation in a sports organization accounts for the prevention of incident functional disability (Table 6). As mentioned above, frequency of meeting friends was then added to the covariates in Model 1 as a measure of social networks. The HR for the EA group dropped slightly from 1.29 (1.02–1.64) to 1.27 (1.00–1.61), and significance disappeared. The HR for the S group was also somewhat attenuated from 1.65(1.33–2.04) to 1.60(1.29–1.98), but the 95% confidence intervals overlapped, and we cannot say that these estimates are statistically different. Addition of either measure of social support resulted in almost no change in the HR for the EA group and the S group.

**Table 6:** Adjusted hazard ratios (95% CI) for incident functional disability by social relations.

	Model 1a)	Model 2b)	Model 3c)	Model 4d)	Model 5e)	Model 6f)
Active Participant (AP)	1.00	1.00	1.00	1.00	1.00	1.00
Exercise Alone (EA)	1.29(1.02–1.64)	1.27(1.00–1.61)	1.29(1.02–1.64)	1.29(1.02–1.63)	1.28(1.01–1.63)	1.30(1.03–1.65)
Passive Participant (PP)	1.16(0.76–1.77)	1.15(0.76–1.76)	1.15(0.75–1.75)	1.15(0.76–1.75)	1.14(0.75–1.74)	1.18(0.77–1.79)
Sedentary (S)	1.65(1.33–2.04)	1.60(1.29–1.98)	1.64(1.33–2.03)	1.63(1.32–2.02)	1.63(1.32–2.02)	1.64(1.33–2.03)

a) Model 1 is adjusted for age, sex, annual equivalent income, educational attainment, marital status, occupation status, self-reported medical conditions, depression, smoking, and alcohol consumption.

b) Model 2 is adjusted for the covariates in Model 1 plus frequency of meeting friends.

c) Model 3 is adjusted for the covariates in Model 1 plus receiving emotional support.

d) Model 4 is adjusted for the covariates in Model 1 plus providing emotional support.

e) Model 5 is adjusted for the covariates in Model 1 plus receiving instrumental support.

f) Model 6 is adjusted for the covariates in Model 1 plus providing instrumental support.

## Discussion

In the present study, we tested incident functional disability by performance of exercise and participation in a sports organization. The HR of incident functional disability was significantly higher for the “Inactive” groups than the “Exerciser” groups. Regarding participation in a sports organization, the risk of incident functional disability was significantly higher in the “Non-participant” groups compared to the “Participant” groups. Furthermore, the HR was significantly higher for the EA group than the AP group. It was reported that participation in social activities leads to a decrease in the risk of functional decline, [7] and incident disability, [18]. These past studies suggest that one such social activity, participation in a sports organization, may lead to incident functional disability prevention. The results of the present study agreed with this hypothesis. Moreover, the results suggest that the health protection effects of participation in a sports organization include not only the physiological effect of exercise, but other effects aside from the exercise itself. Greater amount of exercise has been shown to lead to decreasing risk of functional disability, [6]. There were about twice as many people in the EA group who exercised every day than in the AP group. As a result, there may have been more positive results from exercise in the EA group. It is possible that this effect led to an apparent decrease in the difference in whether or not subjects participated in a sports organization, causing level of participation to be underestimated. This supports the hypothesis that, even when exercise is performed regularly, incident functional disability may be better prevented if they participate in a sports organization than if they do not. When subjects who participated in a sports organization but responded that they “never” exercised were included in the “S” group for analysis, there was no significant difference between the AP group and the PP group. This suggests that participation in a sports organization may help prevent incident functional disability, regardless of frequency of exercise. It remains unclear, however, why there is a difference in incident functional disability with frequency of exercise in those who participate in a sports organization. Future studies are needed to clarify this point.

Next, we tested to see how far health protection effects from participation in a sports organization aside from the effects of exercise could be explained by social relations. When a measure of social networks was added to the covariates in the model, the HR of the EA group dropped, and significant differences disappeared. Using a method for calculating the rate of change from earlier literature “(OR_1_-OR_2_)/(OR_1_-1)*100”, [20], the decrease in HR by social networks was 6.9%. A decrease by 7.7% was also seen for the S group. Only a small fraction of the change was attributable to social networks, but its involvement was nonetheless indicated. An association has also been shown between social networks and decrease in disability, [11], [21]. It is thus conceivable that participation in a sports organization relates more strongly with social networks than private exercise does, and leads to a decrease in incident functional disability. However, the change in HR was minor. In addition, it is possible that social network was not sufficiently evaluated, as frequency of meeting friends was the only measure used.

On the other hand, while social support may also be obtained through participation in a sports organization, adding social support to the covariates had hardly any effect on the HR of the EA group and S group. Group differences in ratio of people with social support were smaller compared to frequency of meeting friends. This suggests that social support has a weaker connection to participation in a sports organization than social networks do. Moreover, in congruence with previous studies, no relationship was seen between incident functional disability and receiving emotional support, [21], [22] or providing social support, [11]. These findings suggest that social support does not explain why participation in a sports organization leads to a decrease in incident functional disability.

The present study has four limitations. The first is that, for people participating in a sports organization, there is no distinction between exercise carried out in the sports organization and private exercise, nor is there any clear distinction among the types of exercise. We acknowledge that people in the “Exercise Alone” category are not necessarily exercising in solitude. Instead, we have used this label to contrast it with people who exercise as part of an organized activity. In future studies, a question should be added to the questionnaires that will enable such distinction. The second limitation is that final analysis was only carried out on about 40% of subjects due to lack of response to the questionnaires and missing variables- therefore it is possible that the results of the present study do not give a complete picture of the region studied. The third limitation is that the sample size in the present study was too small to allow for more detailed analysis of frequency of exercise. Future studies are required to further elucidate the reason why incident functional disability prevention efficacy was greater in the AP group than the EA group and clarify the most preferable frequency of exercise. The fourth limitation is that the only measure used was frequency of meeting friends, though social networks were used to test which aspect of participation in a sports organization serves to prevent incident functional disability. Future studies should consider more aspects of social networks.

In conclusion, tests of the relationship between incident functional disability and 4 groups made up of different combinations of performance of exercise and participation in a sports organization showed a significantly higher HR in the EA group compared to the AP group. This suggests that, even when exercise is performed once a week or more, incident functional disability may be better prevented if the person participates in a sports organization than if they do not. Moreover, when various aspects of social relations were added to the covariates, only inclusion of social networks brought a small reduction in the HR of the EA group and S group. Compared to private exercise, participation in a sports organization correlates more with social networks, which may lead to a small decrease in incident functional disability.
